# Clinical Outcome of Dental Implants after Maxillary Sinus Augmentation with and without Bone Grafting: A Retrospective Evaluation

**DOI:** 10.3390/ma14102479

**Published:** 2021-05-11

**Authors:** Gianluca Martino Tartaglia, Pier Paolo Poli, Stephen Thaddeus Connelly, Carlo Maiorana, Davide Farronato, Silvio Taschieri

**Affiliations:** 1 Department of Biomedical, Surgical and Dental Sciences, University of Milan, 20122 Milan, Italy; gianluca.tartaglia@unimi.it (G.M.T.); carlo.maiorana@unimi.it (C.M.); silvio.taschieri@unimi.it (S.T.); 2 Fondazione IRCCS Cà Granda, Ospedale Maggiore Policlinico, 20122 Milan, Italy; 3 San Francisco Veterans Affairs Health Care System, Department of Oral & Maxillofacial Surgery, University of California, San Francisco, CA 94143, USA; stephen.connelly@ucsf.edu; 4 Department of Medicine and Surgery, School of Dentistry, University of Insubria, 21100 Varese, Italy; davide.farronato@uninsubria.it; 5 Dental Clinic, IRCCS Istituto Ortopedico Galeazzi, 20161 Milan, Italy

**Keywords:** bone substitutes, dental implants, edentulism, grafting materials, sinus augmentation, survival analysis

## Abstract

(1) Background: The purpose of the present study was to retrospectively evaluate and compare the outcome of two sinus augmentation grafting protocols using a xenograft or blood clot alone over a 72-month follow-up. (2) Methods: Patients who received simultaneous lateral sinus floor augmentation and implant placement were included. Subjects were divided into two groups according to the grafting material, namely xenograft or blood clot, and into sub-groups based on the residual alveolar bone height (RABH) below the maxillary sinus, namely 4 to 6 mm or >6 mm. Kaplan–Meier survival estimates were calculated for each material group and for each sub-group at 1, 3, and 6 years. (3) Results: In total, 289 implants inserted in 136 patients with a one-stage procedure were considered. A total of 35 failures were registered. Overall survival rates were 94.2% for xenograft and 85.9% for blood clot alone at 1 year, 91.1% and 81.6% at 3 years, and 91.1% and 78.7% at 6 years. (4) Conclusions: In patients with 4–6 mm RABH, graftless interventions exploiting blood clot alone were not as successful as those using xenograft. When the RABH is low, sinus floor augmentation associated with grafting materials should be preferred whenever possible.

## 1. Introduction

Despite the overall decrease in tooth loss observed in the last decades, the rehabilitation of patients with partial edentulism is the most frequent service provided by dental professionals both in terms of incidence and prevalence [1]. There is a strong consensus within the dental professional community that prostheses supported by osseointegrated implants are the treatment of choice to replace an edentulous span of one or several missing teeth, with well-demonstrated advantages in maintaining physical and mental well-being [2]. In some cases, this clinical goal could not be reached without performing bone augmentation procedures to provide enough hard tissue to support the osseointegrated implants.

Generally speaking, alveolar bone healing after tooth loss is unpredictable, ranging from near-complete maintenance of the alveolar bone to total resorption down to the basal bone, thus limiting the possibility of implant insertion. Different techniques have been introduced as early as the 1980s to avoid bone healing with anatomical restrictions [3,4]. Focusing on the posterior maxilla, during the last four decades researchers, have applied different protocols to optimize the bone volume with different sinus augmentation procedures, materials, and timing for implant surgery [5,6]. Furthermore, short implants measuring less than 8 mm in length as well as zygomatic implants have been successfully used as alternatives to sinus augmentation procedures [7,8].

Today, the variety of techniques have been consolidated and simplified, and two main procedures can be recognized: the lateral antrostomy and the crestal approach, with simultaneous single-stage surgery or delayed two-stages implant insertion [3,9,10,11]. The lateral antrostomy involves the elevation of the Schneiderian membrane through the preparation of a window in the lateral wall of the maxillary sinus. The crestal approach involves the creation of an osteotomy for the selected implants in order to elevate the sinus membrane passing through the same surgical opening, thus resulting in a less invasive method. The osteotomy can be prepared by means of different devices, including compressive tapered manual or pneumatic osteotomes, sinus lift crestal bur kits, piezosurgery, and osseodensification bur kits, amongst others. Both lateral and crestal approaches allow the space beneath the membrane to be grafted using several different materials or left to heal spontaneously with the blood clot alone [4,9,10,12,13,14,15,16,17].

The factors that contribute to the clinical selection of a particular sinus augmentation technique for a specific patient include the characteristic of the native alveolar bone (height, width, density) and whether or not primary implant stability can be obtained [18]. Initially, a minimum of 5 mm of residual alveolar bone height (RABH) was suggested for simultaneous implant placement, but more recent evidence supports the extension of this parameter to include patients with a 2–4 mm RABH [4,14,17,19,20,21]. Bone blocks or chips of autologous, allogenic, xenogenic, or alloplastic material with or without the application of biologically active proteins such as bone morphogenetic protein and platelet-derived growth factor can be used in conjunction with the above-mentioned approaches [5,9,22]. More recently, several researchers have proposed to perform crestal sinus augmentation without grafting material, leaving the blood clot only and the natural healing processes [3,4,12,17,23,24,25].

The first reports describing maxillary sinus floor elevation with grafting materials emerged during the 1980s by Boyne [26] and Tatum [27], while the idea of a graftless approach emerged in the early 1990s with the first experimental work by Boyne [28]. The technique was later described by Ellergaard et al. with the idea that the sinus membrane is left resting on the installed protruding implants, creating a space filled with blood, forming around and between the implants [29]. The newly formed bone is seen around the upper part of the implants protruding up into the sinus cavity. The procedure underwent slight modifications in the following years [30,31] and is used today with generally high reported success rates [4]. Nonetheless, the sinus augmentation technique, the grafting materials, and the timing of implant placement can differently impact bone remodeling and scar contraction characteristics as observed at follow-up [9,32,33].

In view of the aforesaid, the present retrospective study aimed to describe and evaluate the 72-months follow-up of implants placed in the maxillary arch of patients who required a sinus floor elevation surgery. The effect of different RABH and the use of graft or graftless procedures were assessed following a single-stage surgical approach with delayed implant loading. Based on the available literature and previous studies, we expected to obtain generally high survival rates for both tested procedures, with and without grafting material, and evaluate the additional influence of the RABH on the final outcome.

## 2. Materials and Methods

### 2.1. Patient Selection and Study Design

Patients included in the present retrospective evaluation were selected from the oral hygiene maintenance program database of a private clinic (Clinica SST, Segrate, Milano, Italy). All subjects who received simultaneous lateral sinus floor elevation and implant placement from the same private practice between January 1997 to December 2016 were assessed. 

Specific inclusion criteria were as follows: (1) patients of both sexes, older than 18 years, with impaired esthetic and stomatognathic function, per patient report; (2) partially or completely edentulous posterior maxilla; (3) presence of teeth with an unfavorable long-term prognosis; to include one or a combination of the following: (a) tooth mobility with a Modified Miller Index ≥ 3 [34]; (b) residual coronal tooth structure with a tooth restorability index value ≤ 1, (no residual coronal dentine for restoration) [35]; (4) inadequate RABH in the distal maxillary area from the premolar teeth on one or both sides for the placement of at least 10 mm long and 4 mm wide implants without damaging the sinus floor; (5) patient self-report to be in general good health without severe medical or psychological conditions without any pathological findings within the sinuses seen by cone-beam computerized tomography (CBCT) before the surgery; (6) no pregnant or lactating women. Any medical conditions reported by an anesthesiologist or cardiologist contraindicating the surgery were also assessed. 

According to their anamnestic track, patients who reported alcohol or tobacco use were classified as follows: “alcohol consumers” were those who consumed more than half a liter/day of generic alcoholic beverages continuously for at least 1 year, while “smokers” were all patients who declared to be habitual tobacco users [36].

Patients included in the study were recruited at a regularly scheduled oral hygiene maintenance visit where they were informed about the details of the study. Once they understood the purpose of the investigation and agreed to take part, they signed a written consent form. 

The study protocol was conducted according to the STROBE guidelines. All principles in accordance with the Helsinki Declaration for medical research involving human subjects and Italian Law were followed. The Institutional Review Board (SST Clinic, Segrate, Milan, Italy, identification code: IRB02–2005 Doc. MQ 03 AL 03) approved the study protocol.

Pre-surgical examination, orthopantomographs, CBCT scans, and diagnostic study models were obtained from the patient chart. 

All patients and their tracks were evaluated by an independent surgeon who was not involved in the original surgical and prosthetic procedures.

### 2.2. Surgical and Prosthetic Procedures

**Figure 1:**
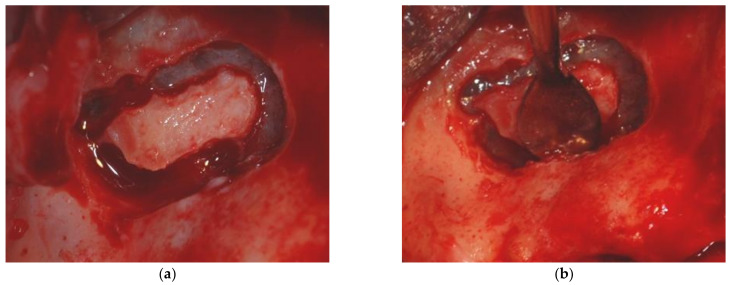
Maxillary sinus floor elevation (**a**) lateral antrostomy; (**b**) careful elevation of the Schneiderian membrane.

The sinus floor augmentation procedure was performed with either a xenograft (Bio-Oss^®^, 1–2 mm large granules, Geistlich Pharma AG, Wolhusen, Switzerland) or the blood clot alone, as further detailed. At the time of intervention, the choice of grafting material/no grafting material was based on the surgeon experience; therefore, no randomization was performed. Sinus floor elevations were carried out as part of the overall oral rehabilitation plan and had been previously determined and agreed on between patients and clinicians. A full-thickness flap was elevated following a crestal incision, with or without mesial and distal releasing incisions extending well up into the buccal fold distant from the implant site. The lateral antrostomy was prepared with a tungsten carbide rose-head bur followed by a small round diamond bur on a low-speed straight handpiece under sterile saline irrigation. The sinus membrane was inspected for tears in all cases (Figure 1).

If a tear was encountered, it was repaired following the classification and techniques described by Vlassis and Fugazzotto [37]. The augmentation procedure was completed, and the grafting material, if planned, was placed concomitantly with the placement of endosseous implants. The surgical flaps were then sutured after implant placement and an orthopantomograph was obtained for all patients.

The procedure was used for single dental replacements, extended dental gaps, or implant-supported full-arch rehabilitations. Bone augmentation procedures were divided according to the graft material (xenograft or blood clot alone) and the RABH (between 4 and 6 mm, and higher than 6 mm). RABH was evaluated on CBCT scans before the surgery. All implants were positioned 1 mm up from the bone level of the alveolar process. The length of the implant portion protruding into the sinus was measured with a calibrated periodontal probe. The implant length (10 or 12 mm) was chosen, taking into consideration the protruding implant length (≤50%) and the crown to implant ratio (≤1). For all rehabilitations, the same implant was used, namely a screw-shaped, acid-etched, rough-surfaced fixture (Titanmed, Milde Implants, Bergamo, Italy).

The sizes and shapes of the individual osteotomies varied from patient to patient and depended on local anatomic considerations. The osteotomies for the implants required the consecutive use of drills of increasing diameter. Drilling was performed by exerting slight intermittent pressure under abundant irrigation with saline solution.

All implants were placed at the time of lateral sinus floor augmentation, maintaining at least 1.5 mm of buccal bone thickness at the buccal and palatal aspects. Implant placement followed standard protocols according to the manufacturer’s instructions [38,39]. To achieve a higher final torque before the ultimate seating of the implant, the countersinking was omitted following under-preparation of the implant osteotomy. The maximum insertion torque, considered as the peak insertion torque reached in the final stage of implant placement into the prepared site was measured with the help of a manual torque wrench.

All flaps were sutured with a 5.0 monofilament suture (Ethicon Inc., Somerville, NJ, USA) to achieve passive primary closure. 

Medications prescribed for postoperative management included chlorhexidine rinses twice a day for 21 days, amoxicillin 1000 mg/two times a day (enteric-coated erythromycin 400 mg three times a day was used for penicillin-sensitive patients), ibuprofen 600 mg/two times a day (paracetamol and codeine three times a day were used for non-steroid anti-inflammatory-sensitive patients), and omeprazole to prevent upper gastrointestinal bleeding in patients at risk.

Implants were not loaded at least 4 months after their placement. Patients were not allowed to use any removable prostheses before the suture removal visit 10 to 12 days postoperatively. At that time, removable prostheses were adjusted, relined, and placed for cosmetic purposes only. Provisional resin restorations were delivered in all cases before final restorations. The final occlusion adjustments were made during maximum voluntary clench and mandibular excursions under contraction.

### 2.3. Patients Follow-Up Assessments

Ideally, all patients underwent postoperative maintenance visits at 6-month intervals consisting of dental hygiene and plaque control instruction. Realistically, not all patients attended these scheduled treatments; however, all patients were seen at least twice per year.

Implants inserted in either grafted or non-grafted sinuses were classified as successful if the fixture was maintained in its place without pain, suppuration, swelling, mobility, discomfort, ongoing pathologic processes, signs of peri-implantitis, neuropathies, or persistent paresthesia [40,41]. According to the latest case-definitions provided in the latest World Workshop on the classification of periodontal and peri-implant diseases and conditions, peri-implantitis was diagnosed in case of presence of inflammatory changes, bleeding on probing, and presence of bone loss starting after the implant was placed in function exceeding 2 mm assessed on intraoral radiographs [41].

Implants were classified as failed in the presence of the above-mentioned signs or symptoms. Additionally, implants were considered as failed if they were removed using a reverse torque tool without causing any damage. If the implant was considered strategic for prosthetic rehabilitation, it was replaced by a new implant after bone regeneration.

A sinus augmentation was deemed successful if sufficient bone was detected using standardized intraoral radiographs at the 6-month dental hygiene recall appointment, and implants placed in the augmented sinus were entirely covered by bone in the absence of sinus disease, as detailed by Dellavia et al. [32]. The sinus augmentation was classified as failed if sufficient bone was not detected using standardized intraoral radiographs at the 6-month dental hygiene recall appointment, and implants placed in the augmented sinus were not entirely covered by bone in the absence of sinus disease.

### 2.4. Statistical Methods

Descriptive statistics were calculated for both grafting material groups, and patient data were summarized accordingly. Student’s *t*-test and Chi-square test, or Fisher exact test when necessary, were used to compare the age and categorical variables between the two grafting material groups. Kaplan–Meier success estimate was performed for patients who were censored or if they had not experienced the end-point of interest at the end of the semi-annual oral hygiene maintenance program recall visits. Calculations were performed at 12, 36, and at 72 months, comparing the grafting material groups (xenograft versus blood clot alone) and the RABH sub-groups (4–6 mm versus > 6 mm). 

To account for possible non-independence of multiple observations from one patient and considering that in some groups there were small numbers of events, cluster robust standard errors were used in all Cox Regression analyses (patients were the cluster variable). Hazard ratio (HR) and confidence intervals (95% CI) were calculated. All analyses were performed using STATA 14.2 (StataCorp, College Station, TX). A two-sided *p*-value < 0.05 was considered statistically significant.

## 3. Results

Overall, 136 Caucasian patients (59 females and 77 males; mean age ± standard deviation: 61 ± 11 years; age range: 25 to 85 years) were considered for this study. Table 1 presents the demographic characteristics of each group.

The majority of patients underwent this procedure as part of the rehabilitation of extended dental gaps, followed by single tooth replacement and implant-supported full-arch rehabilitations (Table 2).

All patients were free from acute periodontal inflammation and chronic inflammatory flares at the time of surgery. There were no differences in age, sex, smoking, or alcohol consumption distributions between the types of grafting material groups.

A total of 289 implants measuring 10 and 12 mm in length 4 or 5 mm in width were placed in 162 grafted sinuses. In detail, most were 4 mm wide (223 out of 289, 77.16%) and 10 mm long (218 out of 289, 75.43%). Irrespective of the implant size, all placed implants reached an insertion torque ≥ 35 Ncm at the time of insertion, measured with the aid of a manual torque wrench. Xenograft was used in 64 patients, corresponding to 80 sinuses, while 72 patients received a graftless procedure, corresponding to 82 sinuses. The median follow-up was 43 months (range: 3–126 months). 

Irrespective of the RABH, a total of 35 failures were registered; nine failures occurred in patients undergoing full-arch rehabilitation, 19 in patients undergoing rehabilitation of extended gaps, and seven in patients undergoing replacement of single teeth (Table 2). Early failures included five implants that were not osseointegrated at the time of uncovering procedure, and seven implants that lost osseointegration probably due to overload within the first 12 months of loading. The remaining implants showed bleeding on probing and progressive bone loss due to peri-implantitis exceeding 2 mm following the first year of function. No significant differences in number of failures between the three types of dental treatments were found comparing the two grafting material groups (Chi-square test, *p*-value > 0.05). The rest of the implants showed healthy peri-implant mucosa associated with marginal bone loss < 2 mm during the entire follow-up period.

**Figure 2:**
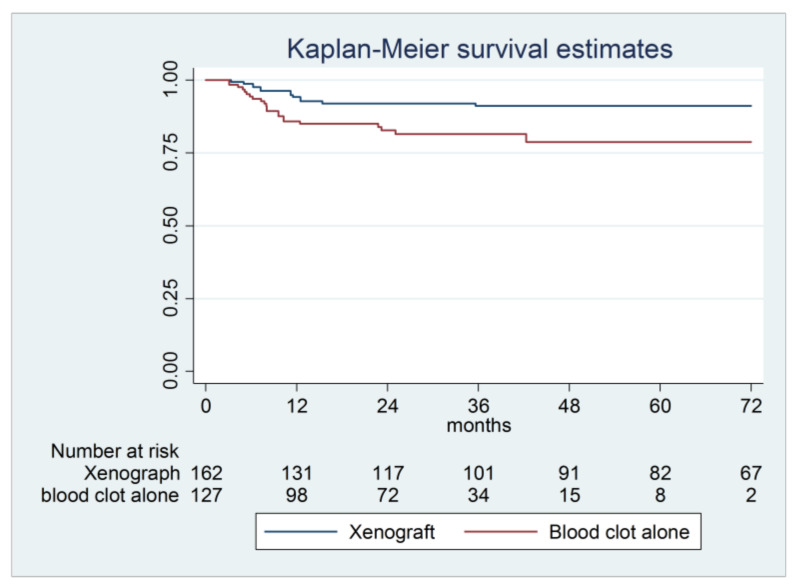
Kaplan–Meier survival curve of implants positioned in maxillary sinuses. The xenograft group had better percentage of survival (95% CI 1.39-6.77, *p*-value 0.006).

Overall, implant success rates ranged from 94.2% for xenograft at 12 months to 78.7% for blood clot alone at 72 months. The graftless procedure showed a higher risk of failure compared to the xenograft group; most failures were reported in the first 12 months of follow-up (Table 3, Figure 2).

**Figure 3:**
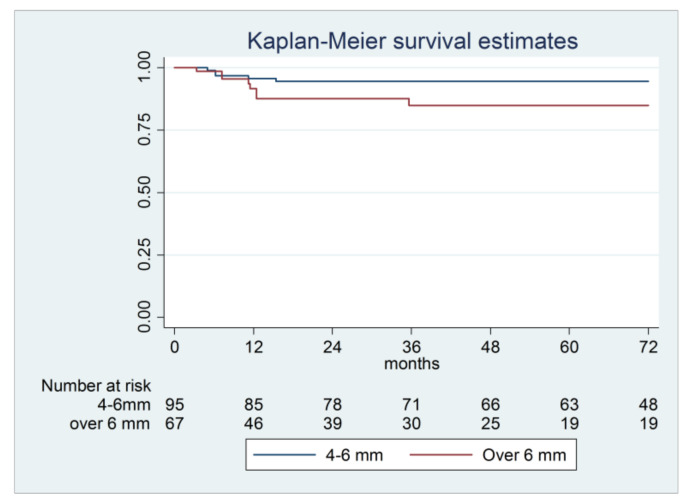
Kaplan–Meier survival curve of implants positioned in maxillary sinuses using xenograft subdivided according to residual alveolar bone before surgery (4–6 mm and over 6 mm) (95% CI 0.72–9.79, *p*-value 0.143).

When considering the two RABH sub-groups in the xenograft group, the lower success rates were observed for the higher RABH, but the difference was not significant (*p* = 0.143); most failures in this sub-group were reported in the first 12 months of follow-up (Table 3, Figure 3).

**Figure 4:**
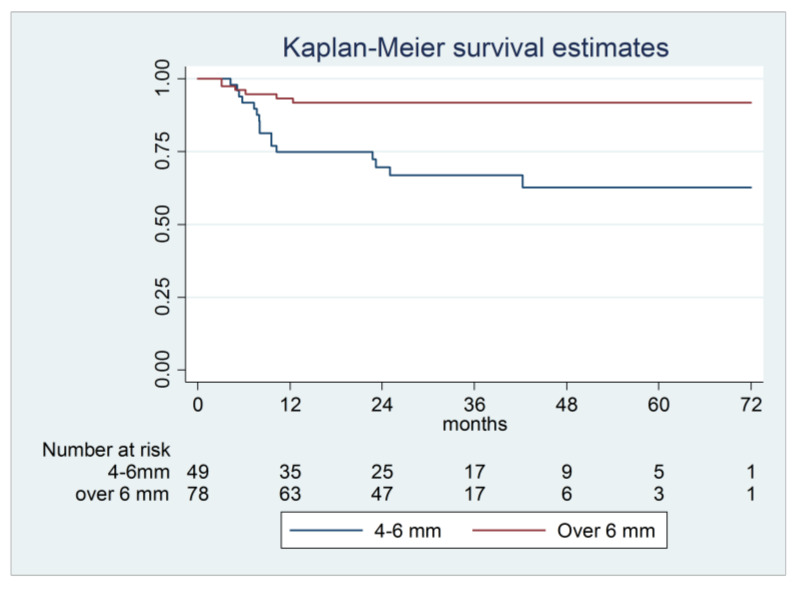
Kaplan–Meier survival curve of implants positioned in maxillary sinuses using blood clot alone subdivided according to residual alveolar bone before surgery (4–6 mm and over 6 mm) (95% CI 0.08–0.66, *p*-value 0.006).

For the graftless procedure, a lower success rate in the 4–6 mm RABH sub-group was observed, with a significantly higher risk of failure compared with the > 6 mm RABH sub-group (*p* = 0.006). Most failures in the 4–6 mm sub-group were reported in the first 12 months of follow-up (Table 3, Figure 4).

Hazard ratio, calculated by Cox regression with cluster robust standard errors for all treatment groups, and the total number of failures in the analyzed follow-up time are reported in Table 3. In general, the blood clot alone showed a higher risk of failure compared to the xenograft; the effect was particularly important for patients with a thinner RABH. The increased risk of implant failure in patients with lower RABH after the graftless procedure was confirmed (*p* = 0.001) by comparison between the same RABH sub-groups of different material groups, which further demonstrated the impact of RABH in the graftless procedure, but not in the xenograft group. The sub-group of RABH > 6 mm showed a higher risk of failure when using the xenograft compared to the blood clot alone, but the results were not statistically significant (*p* = 0.505)

## 4. Discussion

The main finding of the current retrospective data analysis was that, in general, graftless one-stage sinus floor elevation interventions with implant placement in patients with RABH between 4 and 6 mm were not as successful as those using xenograft. 

This outcome contrasts with that reported by a number of recent studies with high implant survival rates of graftless sinus lifting procedure. On several occasions in those studies, data were not separated into distinct thickness classes, and only pooled values were reported, making this assessment difficult [4,10,12,19,23,42]. Thus, dividing RABH into subgroups allowed us to observe the differences between two graft groups and conclude that when the RABH is low, sinus floor augmentation with grafting materials should be preferred whenever possible.

A recent study on RABH ranging from 2 to 8 mm below the maxillary sinus reported that the sinus elevation technique without employing grafting material worked independently of the thickness of the residual alveolar bone, with overall 95.9% implant survival after 8 years [43].

Similarly, with RABH higher than 3 mm, some investigations reported that the use of grafting material gave no significant advantage relative to blood clot alone. In a group of 48 patients with a RABH between 6 and 8 mm, Marković et al. assessed the 2-year implant success rate of a single-stage procedure comparing the effect of various graft materials or blood clot alone. The success rate was 100% [12]. 

The same results were described by Altintas et al. in a 6-month follow-up evaluation of patients with a 4–6 mm RABH for both graft and graftless procedures [44]. A slightly higher survival rate for graftless procedures compared to groups using grafts were published by Cara-Fuentes et al., who followed-up for one year two groups of patients who were submitted to a single-stage procedure [45]. Before treatment, the patients had a RABH between 4 and 7 mm. The success rate was 93% for the 38 implants with xenograft and 97% for the 38 graftless implants. Similarly, in a study conducted by Nedir et al., success rates of 94.1% and 90% were reported for graftless and grafting techniques, respectively [46]. 

Some studies report slightly higher success rates for procedures performed with a graftless approach compared to grafted sinuses, e.g., success rates of 96.4% and 100% were observed by Borges et al. [47] and of 95.0% and 95.2% by Si et al. [48], respectively.

These literature findings are partially in accordance with the present overall results for sinuses of both RABH sub-groups treated with xenograft, and sinuses with RABH > 6 mm left to heal spontaneously with the blood clot alone. However, in the case of graftless procedures with RABH between 4–6 mm, the observed survival rates of 74.9% (1 year), 66.8% (3 years), and 62.6% (6 years) differed from those reported in the literature. 

The difference in survival rates of the two protocols was statistically significant, with a higher failure risk in the blood clot alone sub-group. It has to be mentioned that the majority of failures in the blood clot group, characterized as implant loss, occurred in the first year. This fact can be partially explained by the single-stage surgical procedure. Riben and Thor performed a sinus floor elevation procedure without grafting and with immediate implant placement in patients with RABH between 1 and 10.25 mm; they followed up 87 implants for a mean of 4.6 years and reported a survival rate of 94.3% with early implant failure recorded in three patients with a 1 mm RABH and in one patient with a 2 mm RABH [17]. These failures were attributed to the limited RABH.

Similar to our observations, less satisfactory results with thinner RABH in graftless augmented sinuses were previously reported. 

For instance, the graftless two-stages procedure made by de Oliveira et al. for 10 patients with an average RABH of 3.2 mm failed to promote the formation of bone with sufficient amount and quality for implant installation [13].

Riben and Thor recently performed an extensive review of sinus elevation interventions where only the blood clot was used [4]. They reported data from two single-stage and 10 two-stage procedures in patients with RABH over 3 mm, with only two studies enrolling patients with RABH thinner than 3 mm. The average follow-up times ranged from 8 to 64 months, and the related success rates ranged from 85.4% to 100%. The lowest reported success rate of 85.4% by Ellegaard et al. [30] is in accordance with our general results for the graftless group at 1-year follow-up (85.5%). On the other hand, when RABH is taken into account in a graftless group, the survival rate of the sub-group with RABH between 4–6 mm was higher than in that reported in the current study.

A long-term study of graftless sinus floor elevation by Si et al. resulted in significantly lower survival of implants placed in RABH ≤ 5 mm (78.9%) compared to RABH ≥ 5 mm (93.5%) [49]. Residual alveolar bone prior to the procedure was the only factor associated with implant failure. 

Similarly, lower survival rates of one-stage ITI^®^ sinus implants inserted into graftless augmented sinus were reported as decreasing from 98.5% at 1 year, to 88.7% at 5 years, and to 79.9% at 10 years [30]. Low survival rates were observed in periodontally compromised patients, with smoking and the number of residual teeth being the most influencing factors. 

The studies and available literature suggest that low initial alveolar residual bone, use of shorter implants (<10 mm), periodontitis, and smoking play important roles in the overall survival rates of implants following graftless sinus augmentation.

Other investigators confirmed that implants placed in more atrophic maxillae, left protruding between 4 and 8 mm into the sinus, fail more [50,51,52]. This is likely due to the insufficient RABH during the slow and long-lasting process of new woven bone formation around the newly placed implant. Accordingly, other authors suggested that it is not the RABH influencing the survival of implants, but rather the implant protrusion into the sinus cavity. The initial theory, namely the higher the membrane is elevated, the more bone would be created, was proven incorrect when Sul et al. showed that there was no difference between 4 mm protruding implants and 8 mm protruding implants regarding new bone height in the sinus [53]. Moreover, the excessive implant protrusion might lead to the tension of the sinus mucosa, destroying its integrity. 

However, even lower survival rates were observed in our study in patients with low RABH than those described in the mentioned publications.

Another aspect to be considered is the timing of implant placement. Indeed, several investigators stated that immediate implant placement can be useful from a mechanical standpoint, as they act as space-maintainers when RABH is low [4,16,44]. Nonetheless, in two-stage graftless procedures, screws can be used to the scope [13].

In the current study, all procedures were performed with a single-stage approach. Graftless implants were 3.06 times more likely to be unsuccessful than those grafted with xenograft. In our clinical experience, the use of graftless procedures was introduced according to the favorable literature reports [3,4,16,17,20,23], but the current outcome is suboptimal. Similarly, the survival rates of blood clot alone presented in this publication are lower than those reported by other authors [4], except by Ellegaard et al. [30], who reported a survival rate of graftless sinuses of roughly 85%. In particular, one of the latest systematic review on the topic reported implant survivals in graftless augmented sinuses ranging from 96% to 100%, concluding that maxillary sinus floor elevation without a graft material seems to be characterized by increased new bone formation and high implant survival rate [54]. It is worthy of note, however, that in both groups, implants have been placed in pristine alveolar bone. Since no sinus complications were observed, it can be speculated that bone loss leading to implant failure initiated coronally and progressed more apically, ultimately reaching the sinus compartment. In other words, peri-implant disease occurred at the expenses of the pristine alveolar bone, with no apparent correlation with the grafting material and the maxillary sinus. Therefore, other reasons rather than the type of grafting material need to be investigated to understand the higher failure rate observed in one group compared to the other. In this respect, it should be reminded that peri-implant disease is a site-specific entity, with multiple local predisposing factors that may have triggered the onset of peri-implantitis and biased this result. In this context, as discussed afterwards, the retrospective nature of the present study constituted a limitation, as clinical data considered negligible during patient rehabilitation may have hidden relevant risk factors associated with peri-implant disease. 

Relatively short healing time had not been shown as a factor influencing implant failures, and similar results can be reached in a short healing period compared to the traditional 6 months [55,56].

Modifications in the surgical protocol should be evaluated especially in sinuses with a reduced RABH, considering the intra-surgical primary implant stability. On the other hand, considering that no episodes of sinusitis that required additional treatment were recorded and being the graftless technique cost-effective, less time-consuming, and less expensive when compared with graft procedure, further studies should be planned in order to improve the percentage of a successful outcome.

The two main limitations of our study are its retrospective design and the lack of randomization. Retrospective studies, unfortunately, may lack clinical information that was considered irrelevant at the time of patient treatment, thus leaving some unexplored areas in the study. The choice of using a xenograft or the blood clot alone was entirely left to the experience of the surgeon; therefore, no true randomization was done. Even if the general characteristics of the two groups did not differ statistically, this limitation should be considered. Another example is represented by the age range of the examined sample, which was rather wide. In this matter, although the wall thickness of the maxillary sinus was not evaluated in the present study, it is reasonable to think that such parameter might change consistently depending on the age of the patient. Interestingly, a study evaluating 430 CBCT scans of 860 sinuses of patients aged between 23 and 86 years, similarly to the present study, observed that the age of the subjects had no statistically significant effect on the lateral wall thickness [57]. In agreement with this finding, Zijderveld and coworkers evaluated the prevalence of different anatomical characteristics in over 100 maxillary sinuses, including thick or think lateral walls [58]. The authors did not find any statistically significant differences with regard to gender or age. Comparably, with the purpose to describe the morphological aspects and variation of the anatomy of the maxillary sinus directly related to implant treatment, Neiva and colleagues found no statistically significant correlation between age and thickness of the lateral wall in Caucasian skulls [59]. 

A general limitation of our study, as well as of several other clinical investigations, is that we did not assess the amount and quality of the newly formed bone inside the grafted maxillary sinuses. These assessments require histological analyses that always involve some additional biological cost. Similarly, the length of the implant protruding into the sinus cavity under the elevated membrane is a strong factor influencing the success of the procedure [43]; however, the implant length/RABH ratio was not taken into account in our follow-up. In this respect, it should be noted that the length of the implant itself might not be a relevant factor in the biological prognosis of the treatment, as shorter dental implants as well as longer dental implants in conjunction with lateral sinus elevation procedures yielded similar marginal bone level changes [60]. This statement has been confirmed in recent controlled studies corroborating the fact that even 6 mm length implants placed in RABH of 5–7 mm showed comparable results in terms of marginal bone resorption compared to regular ≥ 11 mm length implants [61,62]. Following this concept, it is safe to assume that crestal sinus floor elevation should be contemplated to place shorter implants as a more conservative and less invasive approach in case of sinuses with a relatively flat floor anatomy together with a RABH ≥ 5 mm. On the other hand, local contraindications include inadequate RABH (<4–5 mm), crestal bone width not allowing for sufficient primary stability of the implant, and an oblique sinus floor with an inclination of > 45°, which is more prone to perforations [51]. If such requirements are fulfilled, an estimated survival rate of 92.8% is to be expected for implants placed in transalveolarly augmented sinuses, after 3 years in function [63], with increased predictability in cases with a RABH of ≥ 5 mm and with implants of ≥ 8 mm [64].

The implants were used for three main kinds of oral rehabilitations: full arch, extended gaps, and single tooth replacement. Inside each category, additional subgroups can be described, for instance, fixed or removable prosthesis, and position of the replaced single tooth, which should be assessed separately. Actually, the reduced number of implants in some categories prevented further sub-analyses but this should be a goal for future investigations. Additionally, the bone quality was not assessed before the procedure, which could play an important role in early implant failures [56]; therefore, further studies considering all mentioned factors should be conducted. Another aspect that should be briefly discussed is the crown to implant ratio. In the present study, implant length was chosen in order to obtain a crown to implant ratio ≤ 1. This was based on the assumption that higher values may lead to non-axial forces where the crown acts as a lever arm creating a bending moment. The resulting mechanical stress is transferred to the first bone to implant contact, thus increasing the risk of developing marginal bone loss. For such reason, crown to implant ratios ranging between 0.5 and 1 were suggested to prevent crestal bone resorption [65]. However, 35 failures still occurred despite the favorable crown to implant ratio. This finding supports the current evidence, suggesting that crown to implant ratio does not seem to be directly related to increased marginal bone loss and does not represent a biomechanical risk factor for the stability of the prosthesis and for the survival of dental implants [66]. According to the present study, this also favorably applies to implants placed simultaneously with sinus floor elevation procedures with or without the use of grafting materials [67]. Finally, the required sample size was not calculated prior to this retrospective data analysis, and we cannot exclude that the lack of significant differences between the outcome of the two RABH sub-groups of the xenograft group may derive from an insufficient number of patients and implants. However, 95% confidence intervals were reported to offer a more detailed assessment of the results.

## 5. Conclusions

The current study showed that maxillary sinuses with a RABH ≥ 4 mm can be successfully grafted with xenograft using a single-stage procedure. One-staged graftless procedures with blood clot alone and immediate implant placement showed lower success rates and should be used with caution, and only in sinuses with a RABH > 6 mm.

## Figures and Tables

**Table 1:** Demographic characteristics of the patients (age, sex) and analyzed risk factors (smoker, alcohol consumer) in the two treatment groups. No significant differences were found among treatment groups (in all occasions *p* ≥ 0.05).

	Xenograft	Blood Clot	*p*-Value
**N° of patients**	64	72	
**Age** (mean ± SD)	61 ± 12.2	61 ± 11.8	0.344 ^1^
**N° of sinuses**	80	82	
**N° of implants**	162	127	
RABH 4–6 mm	95	49	
RABH > 6 mm	67	78	
**Sex**			
Male N° (%)	39 (60.94%)	38 (52.77%)	0.919 ^2^
**Smokers**	11 (17.18%)	11 (15.28%)	0.090 ^2^
**Alcohol consumers**			
N° (%)	8 (12.5%)	6 (8.33%)	0.425 ^2^

^1^*p* values from Student’s *t* test; ^2^ *p*-value from Chi square test.

**Table 2:** Overview of dental treatment and corresponding implant failures.

	Implant Supported Full-Arch Rehabilitation	Extended Gap Rehabilitation	Single Tooth Replacement
**N° of Patients**	21	82	33
Blood Clot Group	11	44	17
Xenograft Group	10	38	16
**N° of Implants/Failures**			
Blood Clot Group	19-April	88/14	20-April
RABH 4–6 mm	03-June	30-September	13-April
RABH > 6 mm	13-January	May-58	7/0
Xenograft Group	29-May	103/5	30-March
RABH 4–6 mm	18-March	January-56	21-January
RABH > 6 mm	02-November	April-47	02-September

**Table 3:** Overall Kaplan–Meier survival rate, Hazard ratio (HR), and confidence intervals (95% CI) for xenograft and blood clot groups.

		12 Months		36 Months		72 Months				
	TF ^1^	SR ^2^ (%)	95% CI	SR (%)	95% CI	SR (%)	95% CI	HR ^3^	95% CI	*p*-Value
**Xenograft**	13	94.20	89.1–96.9	91.10	85.1–94.8	91.10	85.1–94.8	1		
**Blood Clot**	22	85.90	78.3–91.0	81.60	73.0–87.6	78.70	68.2–86.1	3.06	1.39–6.77	0.006 *
**Xenograft Group**										
RAB 4–6 mm	5	95.70	89.0–98.4	94.60	87.5–97.7	94.60	87.5–97.7	1		
RAB over 6 mm	8	91.70	81.0–96.5	84.90	71.6–92.3	84.90	71.6–92.3	2.65	0.72–9.79	0.143
**Blood clot Group**										
RAB 4–6 mm	16	74.90	60.0–84.9	66.80	50.7–78.6	62.60	45.4–75.7	1		
RAB over 6 mm	6	93.30	84.5–97.2	91.80	82.6–96.2	91.80	82.6–96.2	0.23	0.08–0.66	0.006 *

^1^ Total failures; ^2^ survival rate; ^3^ calculated by Cox regression with cluster robust standard errors for all treatment groups, and the total number of failures in the analyzed follow-up time is reported. Hazard ratios (HR), 95% Cis, and the relevant *p*-values were calculated and compared. The blood clot alone procedures showed a higher risk of failure compared with xenograft procedures. The RAB 4–6 mm sub-group of blood clot only showed a significantly higher risk of failure compared with RAB over 6 mm sub-group (*p* = 0.006); * statistically significant difference.

## Data Availability

No new data were created or analyzed in this study. Data sharing is not applicable to this article. The corresponding author remains available for any further clarification.
